# Probiotic and Vitamin D Ameliorate TNBS-Induced Colitis by Targeting Mucosal Barrier and Neutrophil Infiltration

**DOI:** 10.3390/nu17172719

**Published:** 2025-08-22

**Authors:** Jonathan López-Carrasquillo, Vivianka Y. Ramos-Plaza, Myrella L. Cruz, Bryan M. Rodriguez-Morales, Raphael Sánchez, Pablo López, Gladys Chompré, Caroline B. Appleyard

**Affiliations:** 1 Department of Basic Sciences, Ponce Research Institute, Ponce Health Sciences University, Ponce, PR 00716, USA; jonlopez19@stu.psm.edu (J.L.-C.);; 2 Biology and Biotechnology Department, Pontifical Catholic University of Puerto Rico, Ponce, PR 00716, USA

**Keywords:** inflammatory bowel disease, probiotics, vitamin D, macrophage, tight junction, neutrophil

## Abstract

Background/Objective: Probiotic and vitamin D supplements are widely studied in clinical and animal studies as potential treatments for inflammatory bowel disease. However, their potential synergistic or additive effect in ameliorating colitis development is still poorly understood. The aim of this study was to investigate the potential beneficial enhancement of combining a mixed-strain probiotic with vitamin D supplementation in a colitis animal model. Method: After 5 days of acclimation, C57BL/6 mice received Vivomixx probiotic (at least 1 × 10^9^ Colony-Forming Units) and/or vitamin D (5 IU/g) in drinking water and chow, respectively, for 7 days prior to intracolonic TNBS-induced colitis and until sacrifice. On day 10, animals were sacrificed, and colons were collected to assess colonic damage, cytokine and chemokine expression, total M1 macrophage phenotype, and neutrophil recruitment. Serum and fecal samples were collected to assess vitamin D levels and microbiome composition. Results: Administration of probiotic and vitamin D alone or combined decreased colonic damage and neutrophil recruitment and activity. This effect was associated with an increase in the active form of vitamin D in serum and mucosal barrier integrity. However, administration of probiotics and/or vitamin D did not modulate macrophage infiltration or the M1 pro-inflammatory phenotype. Conclusions: These results suggest that combined probiotic and vitamin D supplementation attenuates TNBS-induced colitis by targeting neutrophil infiltration while enhancing the mucosal barrier. This alternative approach may offer protective potential for IBD management.

## 1. Introduction

Inflammatory bowel diseases (IBD), including Crohn’s disease and ulcerative colitis, are debilitating disorders characterized by chronic inflammation of the gastrointestinal tract. Patients with IBD often experience symptoms such as abdominal pain, blood in the stool, and weight loss [1], which severely impact their quality of life. IBD prevalence in North America is expected to increase to 4 million by 2030 [2], while it currently affects over 6.8 million globally [3]. Current treatments, including corticosteroids, immunosuppressive agents, and biologics, aim to reduce inflammatory activity; however, these therapies are associated with significant side effects and the potential for drug resistance, which can lead to treatment intolerance and discontinuation [4,5]. Thus, there is a critical need for alternative or complementary therapeutic approaches to improve treatment outcomes.

The etiology of IBD is complex and not fully understood, but it is believed to involve a combination of genetic, environmental, and microbial factors [6]. The gut microbiome, a diverse ecosystem of bacteria in constant interaction with the host, plays a crucial role in maintaining gut homeostasis. In IBD, an inappropriate immune response to the gut microbiome results in microbial dysbiosis and activation of inflammatory pathways, thereby exacerbating the disease. Additionally, vitamin D deficiency, a commonly reported environmental factor in IBD patients, has been linked to worsened disease outcomes [7,8,9]. These findings have generated interest in probiotics (live microorganisms that confer health benefits when administered in adequate amounts) and vitamin D supplementation as potential strategies for IBD management.

In IBD, mucosal barrier disruption and abnormal immune cell activity are crucial features that exacerbate the condition. The mucosal barrier is composed of a mucus layer formed by goblet cells and epithelial tight junctions. Together, they form a protective barrier that limits the translocation of luminal microbes to the host tissue. However, loss of the mucosal barrier has been commonly observed in IBD patients and animal models [10,11,12], which can lead to the active recruitment and activation of immune cells. Neutrophils are known to play a critical role in IBD pathogenesis. These innate immune cells are among the first responders during an active inflammatory response in the colon and contribute to tissue damage through the release of reactive oxygen species and neutrophil extracellular traps, which are associated with disease severity [13]. On the other hand, macrophages are key players in IBD progression due to their roles in microbial killing, inflammation, and tissue repair, making them a promising target that could be modulated by probiotics and vitamin D [14,15,16,17]. Macrophages can polarize into different phenotypes, primarily classified as pro-inflammatory (M1) and anti-inflammatory (M2). M1 macrophages are associated with high phagocytic capacity, reactive oxygen species production, and the secretion of pro-inflammatory cytokines, while M2 macrophages are involved in tissue repair, anti-inflammatory cytokine production, and immunoregulation [14]. Decreased neutrophil activity and M1 macrophage polarization may help alleviate inflammation and promote remission in IBD.

Due to the potential role of probiotics and vitamin D as immune modulators, interest has arisen in studying their possible synergistic effects. Their combined beneficial effect has been reviewed by Abboud et al. in multiple conditions, such as schizophrenia, gestational diabetes, type 2 diabetes, coronary heart disease, and polycystic ovarian syndrome, where co-supplementation had greater health benefits than individual treatment by decreasing disease severity, inflammation, antioxidative capacity, and reduced use of health care services [18]. The potential interplay between probiotics and vitamin D arises from observations that probiotics have been shown to increase vitamin D absorption and increase vitamin D receptor (VDR) expression in the colon while reducing colitis severity in animal models [19,20,21]. An important role for the VDR in the mechanism of action of probiotics has been further confirmed using VDR knockout models, where their beneficial effects are abrogated [21]. On the other hand, administration of vitamin D has been observed to repolarize M1 macrophage towards M2 phenotype to decrease colitis severity [15]. However, the potential beneficial effect of combining probiotics and vitamin D to target the mucosal barrier and immune cells remains unknown. Given that both the gut microbiome and vitamin D play important roles in regulating the immune system, this study investigated how combining probiotics with vitamin D affects the gut barrier, neutrophil activity, and macrophage polarization in a colitis animal model. This combined approach represents a potential new strategy for managing IBD. By reducing inflammation and supporting healing through natural and complementary treatments, probiotic and vitamin D co-supplementation may offer meaningful improvement in patient care and quality of life.

## 2. Materials and Methods

### 2.1. Animals

Fifty-two male and female C57BL/6 mice (Animal Research Facilities, PHSU, PR), approximately 9–11 weeks old and weighing 21 ± 1 g, were randomly assigned to 4 treatment groups: colitis + vehicle (Veh; n = 16), colitis + probiotic (Pro; n = 14), colitis + vitamin D (VitD; n = 12), and colitis + probiotic + vitamin D (Pro+VitD; n = 10). For randomization, the number sequence was generated using Microsoft Excel software. The mice were individually housed in a room maintained at 21 °C, with a light/dark cycle alternating every 12 h. Bedding was autoclaved, and the animals had ad libitum access to standard laboratory chow and water. The body weight and food consumption of the animals were monitored. All animal experiments were approved by the Institutional Animal Care and Use Committee at Ponce Health Sciences University (protocol code 2203000926). The number of mice used was calculated using G-Power software (version 3.1), based on preliminary data from our laboratory. Sample size was determined using a power of 80%, an alpha level of 0.05, and an effect size (Cohen’s d) of 1.10.

### 2.2. Treatment Delivery and Colitis Induction

**Figure 1:**
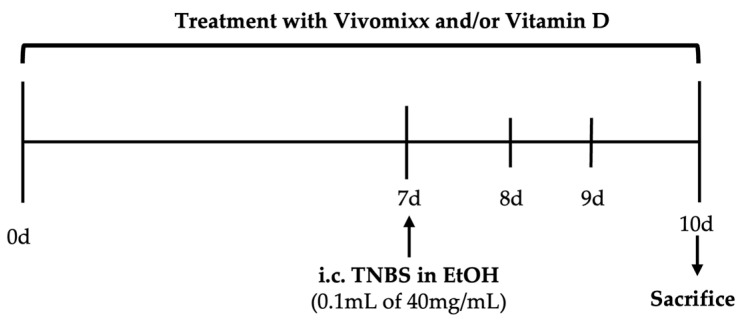
Timeline for treatment delivery and colitis induction. Administration of probiotic Vivomixx (1 × 10^9^ Colony-Forming Units) by drinking water and high vitamin D chow (5 IU/g). Colitis induction was carried out by intracolonic administration of trinitrobenzene sulfonic acid (TNBS) dissolved in ethanol (EtOH).

The probiotic formulation Vivomixx (Mendes S.A., Bordighera, Italy) was generously provided by Dr. De Simon. Vivomixx is a high-potency probiotic formulation containing a blend of eight bacterial strains, primarily lactic acid bacteria and bifidobacteria. The specific strains include *Lactobacillus plantarum*, *Lactobacillus acidophilus*, *Lactobacillus paracasei*, *Lactobacillus delbrueckii* subsp. *Bulgaricus*, *Bifidobacterium infantis*, *Bifidobacterium longum*, *Bifidobacterium breve*, and *Streptococcus thermophilus*. Based on previous studies [22,23], mice in the Pro and Pro+VitD group received 20 billion Colony-Forming Units (CFU) dissolved in 100 mL drinking water and prepared daily (Figure 1). This dose was selected based on prior clinical studies in IBD patients [24], in which a daily intake of 3600 billion CFU was used. This clinical dose has also been used in previous animal studies to guide translational dosing, as 10^9^ CFU is sufficient to achieve colonization [19]. Animals that were not receiving probiotic treatment received maltose dissolved in drinking water. Maltose was also generously provided by Dr. De Simon, since the probiotic Vivomixx is prepared in maltose. Animals in VitD and Pro+VitD were fed with high vitamin D chow (Cat No. TD.10949, Envigo, Madison, WI, USA) at a dose of 5 IU/g. This high-vitamin D chow, formulated from the Teklad 2018 diet with increased cholecalciferol content, has previously been shown to effectively increase serum vitamin D levels and reduce colitis damage without causing hypercalcemia after one week of consumption [25]. Animals that did not receive vitamin D treatment were fed standard laboratory chow Teklad 2018 (Envigo, Madison, WI, USA). Animals received probiotic and high vitamin D treatments starting 7 days before colitis induction and until sacrifice on day 10. Colitis was induced by intracolonic administration of trinitrobenzene sulfonic acid (TNBS, 0.1 mL of 40 mg/mL in 30% ethanol; Cat No. 92822, Sigma Aldrich, St. Louis, MO, USA). Mice were anesthetized with ether, and a 4 cm catheter was inserted through the anus. Mice were held inverted to minimize expulsion of TNBS. Animals were sacrificed 72 h after colitis induction by overdose of pentobarbital. Animals that exhibited significant TNBS leakage at the time of colitis induction or died due to colitis before time of sacrifice were excluded from downstream experiments. Two animals in the Veh group and one in the Pro group exhibited significant leakage. Before completion of the timeline, mortality was observed in Veh (n = 4), Pro (n = 3), VitD (n = 1), and Pro+VitD (n = 3) groups, and these animals were therefore excluded (Supplementary Figure S1). Our study was specifically designed to evaluate the efficacy of probiotic and vitamin D treatments, both individually and in combination, in mitigating inflammation in a well-established murine model of colitis. As such, a healthy control group was not included, given that the primary focus of the research was to determine the most effective protective intervention within the context of an active inflammatory environment.

### 2.3. Sample Collection

At sacrifice, blood was collected by cardiac puncture. The colon was resected and opened to assess macroscopic damage. Proximal and distal sections were longitudinally divided to collect one part for histological analysis and one for molecular analysis. In addition, fecal samples from the colon were collected for molecular analysis. Samples from the brain, liver, and kidney were collected for future experiments. Tissue samples for molecular analysis were snap-frozen in liquid nitrogen and stored at −80 °C. Tissues for histological analysis were fixed in formalin and embedded in paraffin.

### 2.4. Colonic Inflammatory Assessment

Macroscopic and microscopic damage were assessed using previously described methods [26,27]. The macroscopic score is calculated as the sum of scores assessing ulceration (0–10), absence or presence of diarrhea (0–1), absence or presence of stricture (0–1), absence or presence of adhesions (0–2), and bowel wall thickness (millimeters). The microscopic score is calculated as the sum of scores assessing disruption of mucosal architecture (0–3), location of inflammatory cell infiltration (none, 0; in muscularis mucosa, 1; in lamina propria, 2; in serosa, 3), thickness of muscularis propria (<1⁄2 of mucosal thickness, 0; 1⁄2–3⁄4 of mucosal thickness, 1; muscle = mucosal thickness, 2; muscle > mucosal thickness, 3; all muscle), depletion of goblet cells (0–1), and presence of crypt abscess (0–1). Macroscopic and microscopic scoring were performed by a treatment-blinded observer. For each animal, a macroscopic score of the entire colon and a microscopic score for proximal and distal colon were calculated.

### 2.5. Serum Vitamin D Levels

Serum samples collected at sacrifice were used to measure 25-OH vitamin D and 1,25(OH)_3_ using the Mouse/Rat 25-OH Vitamin D ELISA kit (Cat No. VID21-K01; Eagle Biosciences, Nashua, NH) and the Mouse 1,25-Dihydroxyvitamin D3 (DHVD3) ELISA Kit (Cat. No. MBS2602146; MyBioSource, San Diego, CA, USA). The kits were used per the manufacturer’s instructions.

### 2.6. Quantitative PCR for Fecal Probiotic Colonization

To confirm probiotic colonization, fecal samples from day 7 were collected from each mouse cage and stored at −80 °C. DNA extraction was performed using QIamp Fast DNA Stool Mini kit (Cat No. 51604; Qiagen, Germantown, MD, USA) as per the manufacturer’s instruction. The concentration of DNA was quantified by Nanodrop 2000 (ThermoFisher Scientific, Waltham, MA, USA) and was adjusted to 10 ng/mL. Then real-time PCR was conducted to amplify the probiotic strain *S. thermophilus* and 16S ribosomal DNA using IQ SYBR Green Super Mix chemistry (Cat No. 1708882, Biorad, Hercules, CA, USA), as per the manufacturer’s instruction, and specific primers were purchased from Integrated DNA Technologies (IDT, San Diego, CA, USA), which were previously validated [28,29]. The primer sequences were as follows: *S. thermophilus* (forward primer: [5′-TTATTTGAAAGGGGCAATTGCT-3′] and reverse primer [5′-GTGAACTTTCCACTCTCACAC-3′]) and 16S ribosomal DNA (forward primer: [5′-GTGSTGCAYGGYTGTCGTCA-3′] and reverse primer: [5′-ACGTCRTCCMCACCTTCCTC-3′]). For each amplification reaction, 50 ng of DNA template was used. The PCR conditions consisted of an initial denaturation at 95 °C for 3 min, followed by 40 cycles of denaturation at 95 °C for 15 s, annealing at 60 °C for 30 s, and extension at 72 °C for 40 s. The amplification of the 16S ribosomal DNA served as an internal control for normalization. The relative expression levels of the probiotic strain *S. thermophilus* were calculated using the double delta expression method. *S thermophilus* was selected as a representative strain for detecting probiotic colonization due to its high survival rate through gastrointestinal transit and its consistent detectability in fecal samples following administration of Vivomixx [19,30].

### 2.7. Colonic mRNA Extraction and Cytokine Measurement

Distal colon tissue collected at sacrifice was used for molecular analysis. Thirty milligrams of colonic tissue was homogenized in a bullet blender for 5 min in mRNAse-free microcentrifuge tubes filled with beads. RNA was extracted using the RNeasy Mini Kit (Cat No. 74106, Qiagen, Germantown, MD, USA). Then, 1 mg of mRNA was converted to cDNA with the iScript cDNA synthesis kit (Cat No. 1708891, Biorad, Hercules, CA, USA) as per the manufacturer’s instructions. Real-time PCR was performed using IQ SYBR Green Supermix (Cat No. 1708882, Biorad, Hercules, CA, USA) and the following IDT predesign primers: IL-6 (Mm.PT.58.10005566), IL-10 (Mm.PT.58.13531087), or IDT custom primers (see supplementary Table S1). The relative expression level data are reported using the double delta expression method relative to the Veh group.

### 2.8. Immunofluorescence for Macrophage Phenotype and Mucosal Barrier

Immunofluorescence was performed as previously described [31]. Briefly, sections from the distal colon tissue were stained for the macrophage marker F4/80 (Cat No. MCA597R, Biorad, Hercules, CA, USA, 1:100), along with iNOS (Cat No. PA3-030A, Thermo Fisher, Waltham, MA, USA, 1:100) primary antibodies to characterize M1 macrophage infiltration. Secondary antibodies Alexa Fluor 488 goat anti-rat (Cat No. A11006) and Alexa Fluor 555 goat anti-rabbit (Cat No. A21428, Invitrogen, Waltham, MA, USA) were used, respectively. For mucosal barrier assessment, distal colon sections were stained for occludin (Cat No. 71-1500, Invitrogen, Waltham, MA, USA, 1:100) or ZO-1 (Cat No. 50560891, Fisher Scientific, Waltham, MA, USA, 1:100), with secondary antibody Alexa Fluor 555 goat anti-rabbit. The tissue sections were first deparaffinized using a xylene substitute and rehydrated in decreasing alcohol concentrations. Antigen retrieval for macrophages and ZO-1 staining was performed by heating the sections in citrate EDTA buffer (pH 6.2) at 95 °C for 40 min. For occludin staining, antigen retrieval was performed using protease from Streptomyces griseus (Cat No. P5147, Sigma Aldrich, St. Louis, MO, USA). The sections were blocked with anti-goat serum, and PBS washes were performed between steps. Then, tissue sections were incubated with primary antibody overnight. The next day, secondary antibodies were applied for 30 min, followed by PBS washes. DAPI (NucBlue Fixed Cells Stain Probe, Cat No. R37606, Invitrogen, Waltham, MA, USA) was added to stain the nuclei and incubated for 5 min before a final PBS wash. The sections were then mounted with Prolong Gold Antifade Reagent (Cat No. P36934, Invitrogen, Waltham, MA, USA). Images were obtained using an Olympus BX-60 microscope with an X-cite 120Q lamp and photographed with a Nikon DS-FI1 camera or Nikon Confocal Microscope A1 (Ver.4.10) with NIS Elements Software AR 2.22.25 at 400× magnification. For macrophage phenotype assessment, three representative high-power fields (HPFs) per tissue were taken with the same illumination settings and analyzed using ImageJ software (v. 2.14.0/1.54f) by two treatment-blinded observers. The percentage of macrophages expressing F4/80(macrophage marker; green) and iNOS (M1 marker; red) were counted. For occludin and ZO-1 quantification, the integrated fluorescence was measured from three complete villi per HPF, along with the background signal for two HPF per tissue. The background fluorescence was then subtracted from the total villus fluorescence to correct for non-specific staining.

### 2.9. Immunohistochemistry for Vitamin D Receptor (VDR) and Myeloperoxidase (MPO)

Immunohistochemistry was carried out as previously described [19]. Briefly, distal colon sections were deparaffinized in xylene and rehydrated with descending percentage of reagent alcohol. Tissues were then exposed to 3% hydrogen peroxide for 15 min, followed by washing with PBS and antigen retrieval 0.01 M citrate EDTA buffer (pH = 6) at 95 °C for 40 min. After two washes with PBS for 2 min each, protein blocking was performed using normal goat serum for 15 min (Cat no. HK112-9KE, BioGenex, Fremont, CA). Then, overnight incubation was performed with primary antibody VDR (Cat no. ab3508, Abcam, Cambridge, MA, USA, 1:2000), MPO (Cat No. PA5-16672, Invitrogen, Waltham, MA, USA, 1:50), or PBS for negative control on each slide. The next day, slides were washed with PBS for 5 min. A multi-link was used as the secondary antibody for 20 min, followed by PBS wash and Streptavidin Peroxidase incubation for another 20 min (SuperSensitive Link Label IHC Detection System Cat no. #LP000-UCLE, BioGenex, Fremont, CA). For development, one drop of 3,3′ Diaminobencidine (DAB)(Cat no. #HK542-XAKE, BioGenex, Freomont, CA, USA) was applied. Then, tissues were dipped in water, dehydrated in increasing alcohol concentration, and cleared with xylene before mounting using Cytoseal 60 (Cat no. 8310-4, Fisher Scientific, Waltham, MA, USA). Two representative pictures of tissue with mucosa and submucosa at 400× magnification were taken using an Olympus BX-60 microscope for VDR and a Nikon Confocal Microscope A1 (Ver.4.10) with NIS Elements Software for MPO. To assess the amount of colon VDR, color deconvolution and normalization with threshold were performed to measure integrated density. On the other hand, total MPO-positive cells per HPF were counted by two treatment-blinded observers.

### 2.10. Periodic Acid-Schiff (PAS) Staining for Colonic Mucin

Staining for mucin content in distal colon sections was performed using Periodic Acid-Schiff (PAS, Cat. No KTPAS EA, StatLab, McKinney, TX, USA), as per manufacturer’s instructions. Two representative pictures at 400× magnification were taken using a Nikon Confocal Microscope A1 and analyzed in ImageJ software. To assess the amount of mucin in colon sections, color deconvolution and normalization with threshold were performed to measure the percentage of area positive for PAS staining.

### 2.11. Fecal Metagenomic and Microbiome Analysis

At the time of sacrifice, fecal samples were collected directly from the colon, snap-frozen in dry ice, and stored at −80 °C until further processing. DNA was extracted from 200 mg of each sample using the QIAamp Fast DNA Stool Mini Kit (Qiagen, Cat. No. 51604, Germantown, MD, USA), following the manufacturer’s instructions. DNA concentration and purity were assessed using a NanoDrop 2000 spectrophotometer (Thermo Scientific, Waltham, MA, USA). The conserved 16S V3 and V4 region was amplified by polymerase chain reaction. The reaction mixture contained 5 µL of freshly obtained DNA, 12.5 µL of 2x concentrated FastStart master mix from Roche (Roche, Germany), 6.5 µL of molecular grade water (Sigma-Aldrich), and 0.5 µL each of forward and reverse primers [32], yielding a final primer concentration of 0.6µM. The PCR conditions used were as follows: 95 °C for 3 min, followed by 25 cycles of 95 °C for 30 s, 55 °C for 30 s, and 78 °C for 30 s, and a final extension at 72 °C for 5 min. Amplification was confirmed using a Qiagen QIAxcel Advanced instrument with a DNA Screening Cartridge (Qiagen, Germantown, MD, USA). Subsequently, the PCR product was purified using AMPure XP beads (Beckman Coulter), and 8 more cycles were run using Nextera XT indexes to barcode the samples. The barcoded samples were cleaned using Ampure XP beads, and the amplicons were quantified using a Qubit Flex fluorometer with Qubit 1X dsDNA BR Assay Kit (Thermo-Fisher Scientific, Waltham, MA, USA) and screened with the QIAxcel Advanced instrument to ensure proper amplicon size. The resulting libraries were diluted to 20 nM concentration for pooling, further diluted to a final loading concentration of 750 pM and loaded onto a NextSeq 1000 P2 XLEAP-SBS 600 cycles kit, and processed using a NextSeq 1000 instrument. The resulting data was screened to assess the quality of sequences using FastQC software (v.12.1) [33]. Nextera XT adapters were filtered using Trim_Galore! (v0.6.10), and forward and reverse reads were merged using Fastp v0.24.0 [33], with a quality threshold of 30. The Qiime software v.1.9.1 [34] multiple_split_libraries script was used. The sequences were then truncated to a length of 390 bp and reads with more than 1 error and at least 1 N were discarded using Usearch 8. The pick_closed_reference_otus was used to cluster sequences and assign taxonomy using the Greengenes dataset (gg_otus_13_8-release), with 99 percent similarity. Alpha and Beta diversity analyses were produced with the core_diversity_analyses.py from QIIME [35]. Operational Taxonomic Unit (OUT) tables generated by QIIME were uploaded and analyzed using the LefSe (Linear Discriminant Analysis Effect Size) module within the MicrobiomeAnalyst platform [36]. A minimum OUT count of 2 was used to remove singletons, with a minimum prevalence of 20% across samples and a low prevalence filter of 10%. Data were rarefied and scaled using cumulative sum scaling. The significance threshold was set at *p* < 0.05, and the LDA score cutoff was 2.0.

### 2.12. Statistical Analysis

All results are presented as mean ± standard error of the mean and were analyzed using GraphPad Prism v10.2.0 (GraphPad Software, San Diego, CA, USA). A *p* < 0.05 was considered to represent statistical difference. Values more than two standard deviations from the mean were excluded as outliers. To assess statistical differences across treatments, one-way ANOVA with Dunnett’s post-hoc multiple comparisons test was used. A two-way ANOVA with Dunnett’s post hoc test was used to analyze differences in percent weight change across time points. Male and female group analyses were combined, since no changes between sexes were observed.

## 3. Results

### 3.1. Body Weight, Food/Water Intake, and Delivery Confirmation of Probiotic and Vitamin D

**Figure 2:**
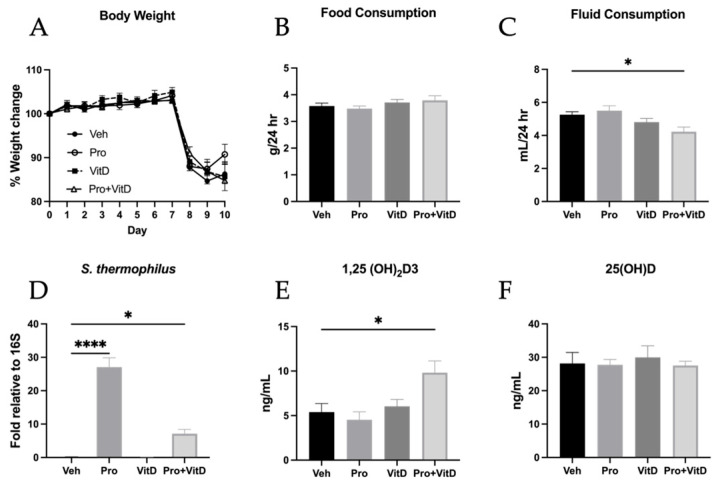
Assessment of animal body weight and treatment delivery. (**A**) Daily body weight changes during the experimental timeline. (**B**) Average daily food intake across treatment groups. (**C**) Average daily fluid intake across treatment groups. (**D**) Fecal detection of Streptococcus thermophilus on day 7 via qPCR, confirming probiotic colonization. (**E**) Serum levels of active form of vitamin D [1,25(OH)_2_D_3_] measured by ELISA. (**F**) Serum levels of inactive vitamin D [25(OH)D] measured by ELISA. Data are presented as mean ± SE (n = 6–14 animals/group; * *p* < 0.05 and **** *p* < 0.0001).

During the 7-day pretreatment phase, no differences in body weight were observed among the treatment groups (Figure 2A). Following TNBS-induced colitis on day 7, all groups exhibited a reduction in body weight until the day of sacrifice on day 10. Notably, only animals in the probiotic-treated group (Pro) showed a trend toward weight recovery, although this was not statistically significant. Because probiotics and vitamin D were administered via ad libitum drinking water and chow, food and water intake were monitored throughout the experiment. Food consumption was observed to be the same across treatments (Figure 2B). However, the animals in the Pro+VitD group consumed significantly less fluid compared to those in the vehicle group (Veh; *p* < 0.05, Figure 2C). To confirm successful probiotic delivery, PCR was performed to detect *Streptococcus thermophilus*, one of the strains in the probiotic mixture. The animals in the Pro and Pro+VitD groups had significantly higher levels of *S. thermophilus* DNA compared to the Veh group (*p* < 0.00001 and *p* < 0.05, respectively; Figure 2D). Serum vitamin D levels were measured using ELISA kits for both the active form (1,25(OH)_2_D_3_) and the inactive form (25(OH)D). The animals in the Pro+VitD group had significantly higher levels of active vitamin D compared to the vehicle group (*p* < 0.05; Figure 2E), whereas no differences in the inactive form were observed between treatment groups (Figure 2F).

### 3.2. Probiotic and Vitamin D Ameliorate TNBS-Induced Colonic Damage

**Figure 3:**
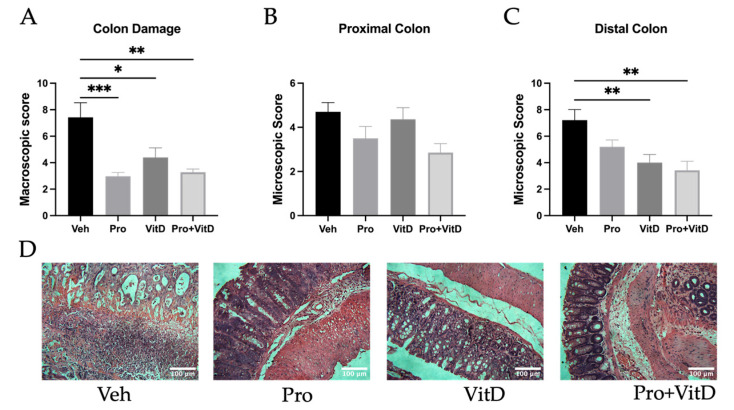
Assessment of probiotic and vitamin D effects on colonic damage. (**A**) Evaluation of colon damage at sacrifice using total macroscopic score. (**B**,**C**) Histological assessment of colonic damage in the proximal and distal colon, respectively. (**D**) Representative histological images (400× magnification; scale bars 100 μm) from the distal colon showing colonic architecture and inflammation in each treatment group: vehicle (Veh), probiotic (Pro), vitamin D (VitD), probiotic + vitamin D (Pro+VitD). Data are presented as mean ± SE (n = 6–11/group; * *p* < 0.05, ** *p* < 0.01 and *** *p* < 0.001).

To evaluate the protective effects of probiotic and vitamin D supplementation on colonic injury, macroscopic damage scores were assessed. Compared to the vehicle group (Veh), the animals in the Pro, VitD, and Pro+VitD groups presented significantly reduced macroscopic colon damage (*p* < 0.0001, *p* < 0.05, and *p* < 0.01, respectively; Figure 3A). At the histological level, proximal and distal colon sections were analyzed for microscopic damage using H&E-stained tissue slides. As expected, the animals in the Veh group showed the highest levels of microscopic damage in both the proximal (Figure 3B) and distal colon (Figure 3C). In the proximal colon, the animals receiving treatment showed a trend toward reduced microscopic damage (*p* = 0.0529); however, these differences did not reach statistical significance. In contrast, in the distal colon, treatment with VitD and Pro+VitD resulted in a significant reduction in microscopic damage compared to Veh (*p* < 0.01). Representative images of histological damage assessment in the distal colon are shown in Figure 3D.

### 3.3. Probiotics and Vitamin D Regulate Colon and Kidney mRNA Transcription

**Figure 4:**
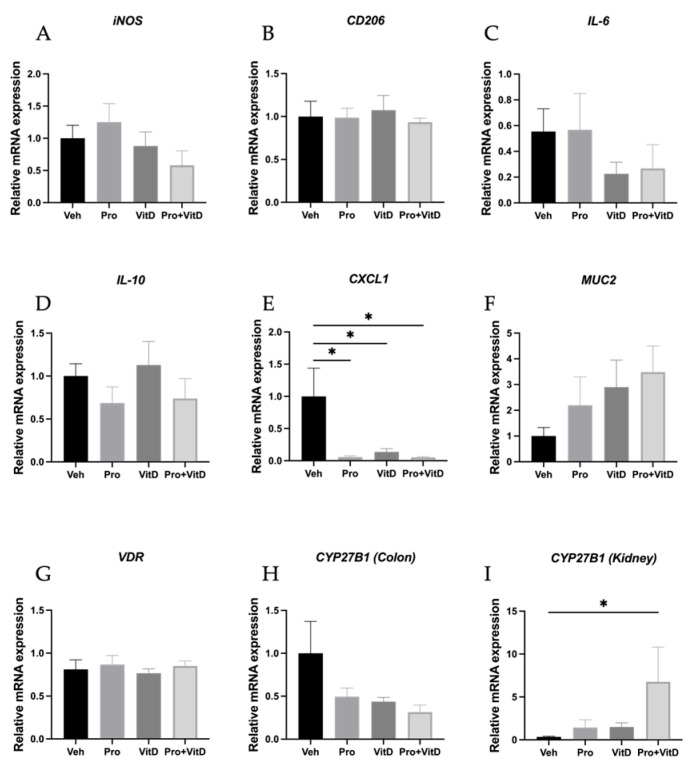
Assessment of colonic and kidney transcript levels. Fold change in distal colon mRNA expression of (**A**) iNOS, (**B**) CD206, (**C**) IL-6, (**D**) IL-10, (**E**) CXCL1, (**F**) MUC2, (**G**) VDR, and (**H**) CYP27B1. (**I**) Fold change in kidney mRNA expression of CYP27B1. Data normalized with beta-actin and calculated using the 2^−∆Ct^ equation. Data are presented as mean ± SE (n = 6–11 animals/group; * *p* < 0.05).

**Figure 5:**
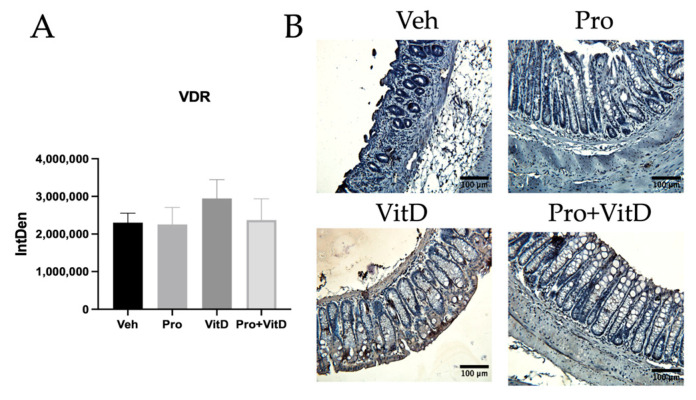
Assessment for colonic vitamin D receptor expression. (**A**) Integrated density of vitamin D receptor (VDR) per high-power field. (**B**) Representative immunohistochemistry images (400× magnification; scale bars 100 μm) from the distal colon showing VDR (brown) with hematoxylin counterstaining in each treatment group: vehicle (Veh), probiotic (Pro), vitamin D (VitD), probiotic + vitamin D (Pro+VitD). Data are presented as mean ± SE (n = 7–11 animals/group).

To investigate the effects of probiotic and vitamin D supplementation on immune activation, vitamin D signaling, and mucosal barrier integrity, gene expression analysis was performed on colon and kidney tissue samples. We first assessed for macrophage polarization markers, including the M1 marker iNOS and the M2 marker CD206, in the distal colon. The mice in the Pro+VitD group showed a trend toward reduced iNOS expression compared to the Veh group, although this difference did not reach statistical significance (Figure 4A). No significant changes in CD206 expression were observed across treatment groups (Figure 4B). Next, we examined the transcription of cytokines involved in inflammatory responses. The pro-inflammatory cytokine IL-6 tended to decrease in both the VitD and Pro+VitD groups, but this was not statistically significant (Figure 4C). Similarly, no significant differences in the anti-inflammatory cytokine IL-10 were observed among groups (Figure 4D). In contrast, the neutrophil-recruiting chemokine CXCL1 was significantly reduced in the Pro, VitD, and Pro+VitD groups compared to Veh (*p* < 0.05; Figure 4E), indicating a potential reduction in neutrophil infiltration in the colon. To assess changes in mucosal barrier function, we measured the expression of MUC2, a key structural component of intestinal mucus. Mice treated with both probiotic and vitamin D (Pro+VitD) showed a trend toward increased MUC2 expression, although this did not reach statistical significance (Figure 4F). Finally, to evaluate vitamin D pathway activation, we measured expression of the VDR and the activating enzyme Cyp27b1 in colon samples. No significant differences were observed in the expression of either VDR (Figure 4G) or Cyp27b1 (*p* = 0.0673 Veh vs. Pro+VitD; Figure 4H) across treatment groups. However, gene expression of Cyp27b1 in kidney tissue was observed to be significantly increased in the Pro+VitD group compared to the vehicle (*p* < 0.05; Figure 4I). To validate VDR gene expression, immunohistochemistry of VDR in distal colon samples was performed. However, only the VitD-treated group exhibited a slight increase in colonic VDR, but this did not reach statistical significance (Figure 5A). Representative pictures of the immunohistochemistry are presented in Figure 5B. To validate our findings, we examined colon samples from a previously established rat TNBS colitis model, also assessed at 3 days post-induction. Consistent with our mouse model, Vivomixx treatment in the rat model did not increase VDR expression at this early timepoint, as shown in Supplementary Figure S2.

### 3.4. Administration of Probiotics and Vitamin D Enhances Mucosal Barrier Integrity

**Figure 6:**
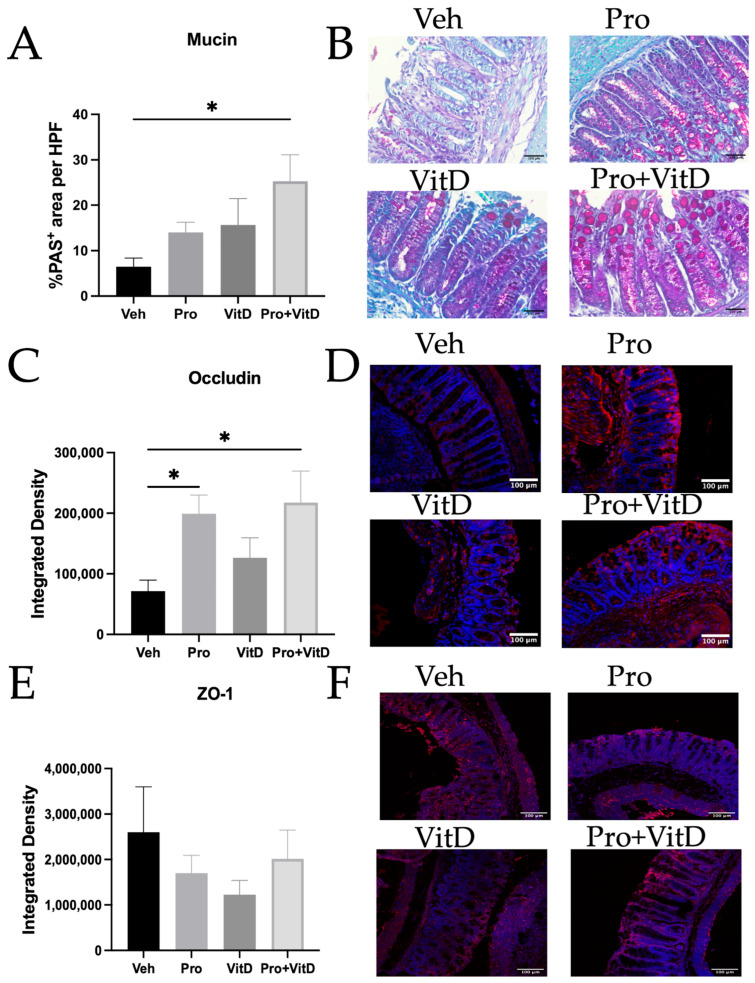
Assessment of probiotic and vitamin D effect on the mucosal barrier in the distal colon. (**A**) Percentage of periodic acid Schiff (PAS)-positive area per high-power field. (**B**) Representative pictures for PAS staining counterstained with hematoxylin (400× magnification and scale bars 100 μm). (**C**) Integrated density of occludin per high-power field. (**D**) Representative pictures of immunofluorescence for occludin (red) and DAPI (blue; 400× magnification and scale bars 100 μm). (**E**) Integrated density of zonula occludens 1 (ZO-1) per high-power field. (**F**) Representative pictures of immunofluorescence for ZO-1 (red) and DAPI (blue; 200× magnification and scale bars 100 μm). Data are presented as mean ± SE (n = 6–10/group; * *p* < 0.05).

To investigate the effects of probiotics and vitamin D on the mucosal barrier, we assessed mucin production and tight junction protein expression in the colon. First, Periodic Acid-Schiff (PAS) staining was used to evaluate the mucin content by quantifying the percentage of PAS-positive area. As shown in Figure 6A,B, the animals in the Pro and VitD groups showed an increase in mucin levels compared to the vehicle group, while the Pro+VitD group exhibited a significantly enhanced effect (*p* < 0.05). Next, we evaluated the expression of tight junction proteins occludin and ZO-1 using immunofluorescence. As shown in Figure 6C,D, occludin expression was significantly increased in the animals treated with Pro and Pro+VitD (*p* < 0.05), indicating improved tight junction integrity. However, no significant differences were observed in ZO-1 expression across treatment groups (Figure 6E,F).

### 3.5. Administration of Probiotic and Vitamin D Reduces Neutrophil Infiltration

**Figure 7:**
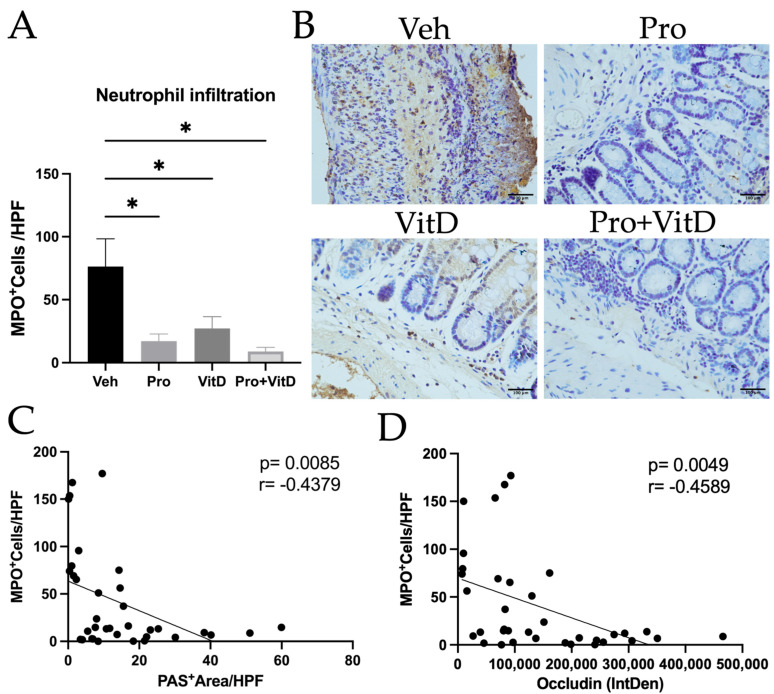
Assessment of the effect of probiotic and vitamin D on neutrophil infiltration in the distal colon. (**A**) Number of MPO-positive cells per high-power field. (**B**) Representative immunohistochemistry images (400× magnification; scale bars 100 μm) from the distal colon showing MPO-positive cells (brown) and counterstained with hematoxylin in each treatment group: vehicle (Veh), probiotic (Pro), vitamin D (VitD), probiotic + vitamin D (Pro+VitD). (**C**) Correlation of PAS-positive area per high-power field with MPO-positive cells per high-power field. (**D**) Correlation of occludin integrated density with MPO-positive cells per high-power field. Data are presented as mean ± SE (n = 6–10/group; * *p* < 0.05).

Neutrophils play a critical role in the innate immune response during the acute phase of colitis. To evaluate the effect of probiotic and vitamin D treatment on neutrophil infiltration, we quantified myeloperoxidase (MPO)-positive cells in the distal colon using immunohistochemistry. MPO was used as a biomarker because this enzyme is abundantly expressed in neutrophil granules, where it has a key role in degranulation and the generation of active oxygen species during an inflammatory response. The results showed that the animals treated with Pro, VitD, or the combination (Pro+VitD) exhibited a significant reduction in MPO-positive cells (*p* < 0.05) compared to the vehicle group, indicating decreased neutrophil infiltration (Figure 7A). Representative pictures of the staining are illustrated in Figure 7B. Interestingly, in Figure 7C,D, neutrophil infiltration was negatively correlated with PAS-positive area of staining (*p* = 0.0085 and r = −0.4379) and occludin expression (*p* = 0.0049 and r = −0.4589), respectively. This suggests that neutrophil infiltration is associated with goblet cell depletion and mucosal barrier integrity.

### 3.6. Probiotic and Vitamin D Treatment Did Not Alter Macrophage Infiltration or M1 Polarization in the Colon

**Figure 8:**
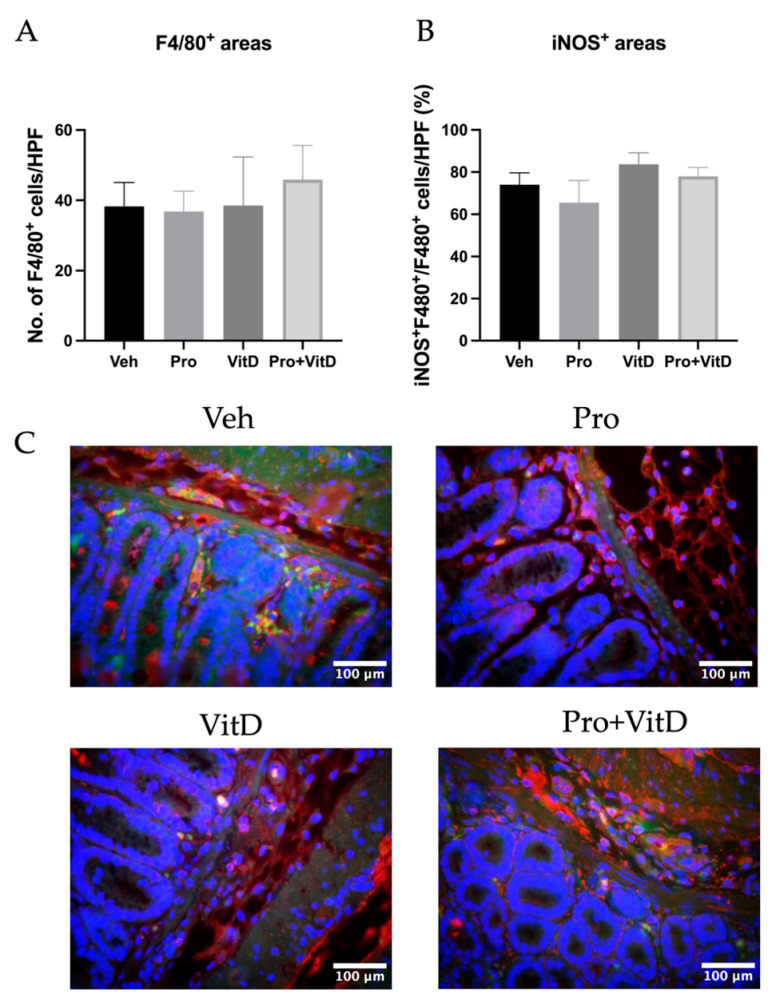
Assessment of macrophage infiltration and polarization. (**A**) Total number of F4/80-positive cells per high-power field. (**B**) Percentage of F4/80 macrophages that are iNOS-positive (M1 macrophages). (**C**) Representative immunofluorescence images (40× magnification; scale bars 100 μm) from the distal colon showing macrophage marker F4/80 (green), M1 polarization marker iNOS (red), and nuclei DAPI (blue) in each treatment group: vehicle (Veh), probiotic (Pro), vitamin D (VitD), probiotic + vitamin D (Pro+VitD). Data are presented as mean ± SE (n = 6–8/group).

To further investigate the effects of probiotic and vitamin D treatment, we performed immunofluorescence staining for F4/80 (a pan-macrophage marker) and iNOS (an M1 macrophage marker) to assess macrophage infiltration and polarization in colonic tissue. First, to evaluate macrophage infiltration, we quantified F4/80-positive cells in the distal colon. However, no significant differences in macrophage numbers were observed across treatment groups (Figure 8A). Next, we assessed the proportion of M1-polarized macrophages by calculating the percentage of F4/80^+^ cells co-expressing iNOS. Similar to infiltration, no significant effect of probiotic or vitamin D treatment on M1 macrophage polarization was observed (Figure 8B). Representative pictures of the immunofluorescence staining are presented in Figure 8C.

### 3.7. Probiotic and Vitamin D Effects on Gut Microbiome Composition

**Figure 9:**
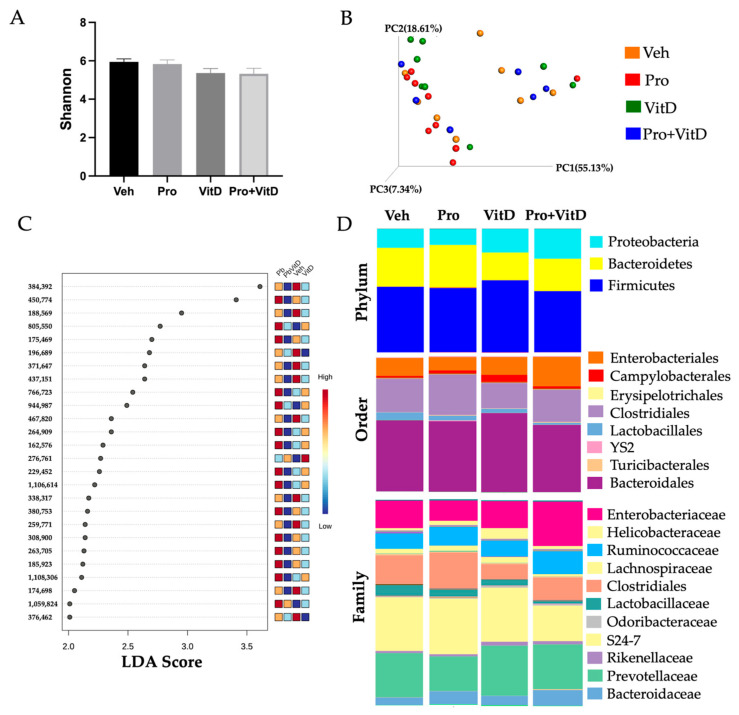
Assessment of fecal microbial composition. (**A**) Alpha and (**B**) beta diversity analysis. (**C**) LefSe Linear Discriminatory Analysis (LDA) score. (**D**) Taxonomic abundance at phylum, order, and family levels in each treatment group: vehicle (Veh), probiotic (Pro), vitamin D (VitD), probiotic + vitamin D (Pro+VitD; n = 6–9/group).

On the day of sacrifice, fecal samples were collected for 16S rRNA sequencing to assess the gut microbial community across treatment groups. Alpha and beta diversity analyses showed no significant differences across treatment groups (Figure 9A,B, *p* > 0.05), indicating similar species richness, evenness, and overall microbial community structure. To further identify microbial markers that discriminated among treatment groups, a LefSe analysis was performed. LefSe identified 26 OTUs with significantly different relative abundance across treatments. The animals in Veh group showed enrichment of taxa primarily within the phyla *Bacteroidetes* and *Firmicutes*, including multiple OTUs classified as belonging to the family S24-7 (OTUs 384392,371647,376462) and unclassified members of the order *Clostridiales* (OTUs 188569, 196689). Additionally, *Lactobacillus* (OTU 338317) was enriched in the Veh group. In contrast, the probiotic-treated (Pro) group exhibited enrichment of several OTUs within Firmicutes, notably unclassified members of *Clostridiales* (e.g., OTUs 450774, 805550, 175469), as well as OTUs assigned to *Clostridiaceae* (OTU 805550), *Peptococcaceae* (OTU 263705), and *Ruminococcaceae* (OTUs 185923, 1108306). Notably, *Streptococcus* (OTU 1059824) and Enterobacteriaceae (OTU 944987) were also significantly enriched in this group. The vitamin D-treated group (VitD) showed selective enrichment of the *Ruminococcaceae* family (e.g., OTU 276761). These differences are illustrated in the LDA bar plot (Figure 9C) and the full list of differentially abundant OTUs is presented in Supplementary Table S2.

In addition, percent abundance was used to see whether differences existed among bacterial taxa across treatments. At phylum level, *Firmicutes*, *Bacteroidetes,* and *Proteobacteria* were the more abundant bacterial taxa (Figure 9D). While not statistically different, animals treated with VitD or Pro+VitD showed a slight decrease in Firmicutes compared to the vehicle group. At the order level, *Lactobacillales* appeared reduced in the Pro, VitD, and Pro+VitD treatments. In contrast, the animals in the VitD group exhibited an increased abundance of *Campylobacterales*, while the Pro group showed decreased *Enterobacteriales*. At the family level, the Pro+VitD group exhibited an increased abundance *of Ruminococcaceae*, while the Pro group had decreased levels of Enterobacteriaceae and increased levels of *Lachnospiraceae*.

## 4. Discussion

In the present study, we investigated the protective potential of combining probiotic and vitamin D supplementation in a TNBS-induced model of acute colitis. Our findings demonstrate that both probiotic and vitamin D treatments, individually or combined, significantly ameliorated colitis development, as evidenced by less macroscopic and histological colitis damage. These effects were associated with enhanced mucosal barrier integrity, reduced neutrophil infiltration, and increased serum levels of the active form of vitamin D.

To our knowledge, only two prior studies have examined the combination of probiotics with vitamin D in a colitis model. One study reported that the combination of krill oil, vitamin D, and *Lactobacillus reuteri* reduced colonic inflammation by downregulating proinflammatory cytokines and upregulating anti-inflammatory cytokines, an effect associated with increased colonic VDR expression [37]. Additionally, a study by Chen et al. demonstrated that combining vitamin D with *Lactobacillus rhamnosus GG* or its main secretory protein, p40, synergistically improved colitis severity by promoting colonic epithelial proliferation, rather than having a protective effect via apoptosis [38]. These effects were abolished in VDR knockout mice. Our study expands upon these previous observations to better understand the impact of combining a mixed-strain probiotic with vitamin D supplementation on their ability to target colonic mucosal barrier integrity and immune cell infiltration and activity.

The beneficial effect of probiotics and vitamin D on decreasing colonic inflammation in this study align with previous research. However, in contrast to prior studies, we did not observe an increase in colonic VDR. Our team has previously shown that administration of Vivomixx probiotic over a 3-month period increases VDR expression in a rat colitis-associated colorectal cancer model, suggesting that VDR upregulation may require prolonged exposure to the probiotic [19]. These results suggest that although probiotics enhance VDR expression under long-term inflammatory conditions, short-term administration during the acute phase of colitis may not be sufficient to induce changes. Additionally, the probiotics were administered ad libitum via drinking water, and the animals were observed to consume less water after colitis induction, potentially limiting the effective dose received during the onset of inflammation.

When examining the effects of vitamin D alone, we observed a slight, non-significant increase in colonic VDR. This finding aligns with that of Chen et al., who also reported that vitamin D treatment alone did not significantly increase VDR expression [38] and suggests that vitamin D supplementation may not be sufficient on its own to robustly increase VDR levels in the context of acute colitis.

Previous research has indicated that probiotics and the gut microbiome can influence vitamin D metabolism and absorption [20,39,40]. Supporting this, we observed that co-administration of probiotic and vitamin D significantly increased renal expression of *cyp27b1*, the enzyme that converts 25(OH)D to its active form, 1,25(OH)_2_D_3_. This finding is consistent with the elevated serum levels of the active form of vitamin D detected in the Pro+VitD group. Interestingly, while *cyp27b1* expression was increased in the kidney, an inverse pattern was observed in the colon, where animals in the colitis vehicle group exhibited elevated colonic *cyp27b1*, and this expression was reduced by probiotic and/or vitamin D treatment. This opposing trend may reflect a local compensatory response to inflammation, as *cyp27b1* is known to be expressed by macrophages [41]. Both M1 and M2 macrophages have been reported to upregulate *cyp27b1* expression and locally convert 25(OH)D to 1,25(OH)_2_D_3_, a process further enhanced by toll-like receptor activation, which promotes calcitriol production to stimulate antimicrobial peptides and facilitate bacterial clearance [41,42]. Therefore, the elevated colonic *cyp27b1* in untreated colitis may represent an attempt to control bacterial infiltration, while its reduction by probiotic and/or vitamin D may indicate decreased immune activation due to improved barrier integrity and reduced inflammation. Taken together, these results suggest that during the early phase of colitis, the probiotic and vitamin D combination may enhance systemic vitamin D metabolism to mitigate the need for local immune driven calcitriol production in colon and potentially activate existing VDR.

The intestine is an organ rich in macrophage numbers and activity due to the constant exposure to foreign antigens. During Crohn’s disease, an increase in macrophage recruitment and polarization toward the M1 pro-inflammatory phenotype are commonly observed [14]. Because of this, there is a need to decrease M1 polarization to reduce colitis development. In this study, probiotic and vitamin D combination did not reduce macrophage infiltration or promote a shift away from M1 polarization, as assessed by real-time PCR for colonic iNOS and CD206, as well as by double immunofluorescence staining for F4/80 and iNOS. These findings expand upon our previous studies, which have shown that Vivomixx multi-strain probiotic administration alone does not reduce M1 macrophage polarization [16,22]. Similarly, other studies have observed that exposure of the Raw264.7 macrophage cell line to conditioned media from the multi-strain Advanced Multi-Billion Dophilus™ probiotic or from Lactobacillus plantarum RS-09 increased phagocytosis and nitric oxide concentration (M1 marker) [43,44]. In contrast, the current results differ from some prior studies which suggests that vitamin D administration can decrease M1 macrophage polarization [15]. A notable distinction and possible explanation, however, is the form of vitamin D and the route of administration. That early study employed intraperitoneal (IP) delivery of calcitriol, whereas we administered cholecalciferol (5 IU/g) orally via chow. The rationale for using oral administration of cholecalciferol was based on its translational value, as it is the most common form of vitamin D used in clinical trials and is widely available commercially. This vitamin D precursor relies on the hydroxylation steps in the liver and kidneys to form calcitriol (active form of vitamin D). Calcitriol is necessary to activate the vitamin D receptor to perform its nuclear transcription functions that can repolarize macrophages toward an M2 phenotype. Administration of calcitriol by intraperitoneal injection bypasses this hydroxylation steps and can directly activate VDR functions, therefore achieving its effects in a shorter period. However, this method of delivery is not commonly used in clinical scenarios due to the risk of hypercalcemia. Therefore, although our model found increased levels of the active form of vitamin D in the co-administration group, achieving higher levels of the active form or a more prolonged treatment period may be necessary to observe an effect on macrophage phenotype.

Importantly, in addition to the form and route of administration, the dose of vitamin D plays a critical role in colitis outcomes. Both vitamin D deficiency and excessive supplementation have been shown to worsen colitis, emphasizing a narrow therapeutic window. For instance, vitamin D deficiency has been observed to impair antimicrobial peptide expression and predispose mice to more severe colitis [45]. On the other hand, high-dose supplementation at 10 IU/g (10,000 IU/kg) has been reported to aggravate colitis and disrupt gut microbial composition, while moderate dosing of 5 IU/g and 0.6 mcg/25 g has conferred protective effects [25,46,47]. These findings suggest that optimal dosing is essential to harness the immunomodulatory effects of vitamin D.

Neutrophils are essential mediators during the acute phase of colitis; however, their activity can also contribute to epithelial injury [48]. During neutrophil infiltration, they can release proteases and reactive oxygen species that can disrupt epithelial tight junctions and mucin layers, exacerbating barrier dysfunction. In our study, we observed that co-administration of probiotic and vitamin D significantly reduced the neutrophil chemoattractant CXCL1 expression and the number of MPO-positive cells in the colon, indicating decreased neutrophil recruitment. Similarly, co-administration of probiotic and vitamin D increases mucin and tight junction, as evidenced by MUC2 (building block of mucin), PAS-positive staining, and occludin expression, suggesting an improved goblet cell function and mucosal barrier. Interestingly, changes in the PAS-positive area and occludin expression correlated with the decrease in neutrophil MPO-positive cells, suggesting that enhanced improvement in tight junction integrity and mucin production may reduce neutrophil activity. Our results align with previous reports showing that vitamin D administration decreased neutrophil recruitment in a DSS animal model of colitis [49]. Similarly, previous studies have observed that activation of VDR enhances mucosal barrier function by regulating the tight junctions occludin, ZO-1 and claudin-2, as well as promoting MUC 2 expression and antimicrobial peptide production [50,51,52]. Therefore, these findings highlight the beneficial effect of combining probiotics and vitamin D to strengthen the mucosal barrier as a potential mechanism to limit excessive neutrophil accumulation and damage.

Disruption of the gut microbial community is commonly recognized as a central contributor to the pathogenesis of IBD [4,53]. To further explore potential mechanisms that contribute to decreased colonic damage, we analyzed fecal microbiota composition across treatment groups. While alpha and beta diversity did not show significant differences between groups, the LefSe analysis revealed distinct microbial signatures associated with each treatment. The directional change in microbial composition suggests that probiotic and vitamin D treatment modulates the gut microbiome, reducing pathobionts (*Proteobacteria* and *Enterobactericiae*) and increasing beneficial commensals (*Streptococcus*, *Lacnospiraceae*, and *Ruminococcaceae*). Within the firmicutes phylum, *Lachnospiraceae* and *Ruminococcaceae* have commonly been observed to be decreased in IBD patients [53,54]. Both families have been previously described as major producers of short-chain fatty acids, such as butyrate, which is known for its critical role in intestinal health [55]. As reviewed by Recharla et al., butyrate acts as a primary energy source for colonocytes, improves tight junction integrity, promotes mucus production, and has anti-inflammatory effects via inhibition of Nf-kB and induction of regulatory T cells [56]. Therefore, the observed increase of *Lacnospiraceae* and *Ruminococcaceae* in response to probiotic and vitamin D may represent a mechanism contributing to the protective effects on epithelial integrity and immune regulation.

While our study findings offer new insights into the potential synergistic effect of probiotics and vitamin D, there are some limitations that should be acknowledged. First, the TNBS-induced colitis model primarily mimics the acute phase of inflammation and may not capture chronic or relapsing features commonly observed in IBD patients. Second, we assessed outcomes at 3 days post TNBS, which may limit insight into long-term treatment effects. Third, although we observed significant changes in neutrophil recruitment, vitamin D levels, and mucosal barrier markers, we did not perform mechanistic or functional assays to directly assess mucosal permeability. Therefore, future studies should incorporate the combination of probiotic and vitamin D in longer-term treatments to further assess their role in chronic and relapsing models, as well as in models of colitis-associated colorectal cancer, to better understand their therapeutic potential. Additionally, studies should explore more molecular pathways that probiotics and vitamin D could synergistically impact, such as immune cell signaling and epithelial repair.

## 5. Conclusions

In conclusion, our findings demonstrate that co-administration of probiotics and vitamin D attenuates TNBS-induced colonic inflammation, primarily by enhancing mucosal barrier integrity, reducing neutrophil infiltration, and increasing serum vitamin D levels. These effects occurred without changes in macrophage infiltration or M1 polarization, suggesting that beneficial effects in the acute phase of colitis are mediated through barrier preservation and innate immune modulation. This study builds on previous observations to support the potential of combining probiotic and vitamin D supplementation as a complementary strategy to promote mucosal healing and remission in inflammatory bowel disease.

## Data Availability

The raw data supporting the conclusions of this article will be made available by the authors on request.

## Supplementary Materials

The following supporting information can be downloaded at https://www.mdpi.com/article/10.3390/nu17172719/s1. Figure S1: Assessment for survival; Figure S2: Assessment for colonic vitamin D receptor in a rat TNBS colitis model.; Table S1: Primers used for real-time q-PCR.

## Abbreviations

The following abbreviations are used in this manuscript:

IBD	Inflammatory bowel disease
VDR	Vitamin D receptor
CFU	Colony-Forming Unit
TNBS	Trinitrobenzene sulfonic acid
EtOH	Ethanol
DNA	Deoxyribonucleic acid
RTPCR	Real-time polymerase chain reaction
RNA	Ribonucleic acid
cDNA	Complementary deoxyribonucleic acid
mRNA	Messenger ribonucleic acid
IL	Interleukin
iNOS	Inducible nitric oxide synthase
ZO-1	Zonula occludens 1
EDTA	Ethylenediaminetetraacetic acid
DAPI	4′,6-diamidino-2-phenylindole
PBS	Phosphate-buffered saline
HPF	High-power field
MPO	Myeloperoxidase
DAB	3,3′-diaminobenzidine
PAS	Periodic Acidic Schiff
ELISA	Enzyme-Linked Immunosorbent Assay
SE	Standard Error of Mean
MUC2	Mucin 2
H&E	Hematoxylin and Eosin
