# Supplementary material for: Probiotic and Vitamin D Ameliorate TNBS-Induced Colitis by Targeting Mucosal Barrier and Neutrophil Infiltration
Source: Nutrients. 2025 Aug 22;17(17):2719. doi: 10.3390/nu17172719 (PMC12430164; doi:10.3390/nu17172719)
Supplement: Supplementary file 1 [file nutrients-17-02719-s001.zip › nutrients-3794870-Supplementary.pdf]

Supplementary Table S1: Primers used for real-time q-PCR.

Genes	Primer Forward (5'-3')	Primer Reverse (5'-3')	Anneling Temperature (°C)	Reference
CD206	CCA TTT ATC ATT CCC TCA GCA AGC	AAA TGT CAC TGG GGT TCC ATC ACT	57	Lingling Li et al. 2020
INOS	ATT CAC AGC TCA TCC GGT ACG	GGA TCT TGA CCA TCA GCT TGC	57	Lingling Li et al. 2020
VDR	CTT CCT AAG AGA CTT CCC GAG AGA	GGC ATT TAT TTA CAG CGG TAC TTG T	57	Xiaoliang Zhang et al. 2015
CYP27B1	AGT GTT GAG ATT GTA CCC TGT G	TAG GGA GAC TAG CGT ATC TTG G	55	Mayara C. Ribeiro et al. 2022
MUC2	CAA GTG ATT GTG TTT CAG GCT C	TGG AGA TGT TCT TGG TGC AG	55	Jay R. Thiagarajah et al. 2014
CXCL1	CCG AAG TCA TAG CCA CAC TCA A	GCA GTC TGT CTT CTT TCT CCG TTA C	57	Sj Shen et al. 2021
B-ACTIN	TGA GAG GGA AAT CGT GCG TGA C	GCT CGT TGC CAA TAG TGA TGA C	57	Xiaoliang Zhang et al. 2015

Supplementary Table S2. List of Discriminative OTUs with Taxonomic Assignment and LDA Score from LEfSe Analysis score.

OTU	Taxonomy	LDA score
**384,392**	k__Bacteria; p__Bacteroidetes; c__Bacteroidia; o__Bacteroidales; f__S24-7; g__; s__	3.61
**450,774**	k__Bacteria; p__Firmicutes; c__Clostridia; o__Clostridiales; f__; g__; s__	3.41
**188,569**	k__Bacteria; p__Firmicutes; c__Clostridia; o__Clostridiales; f__; g__; s__	2.95
**805,550**	k__Bacteria; p__Firmicutes; c__Clostridia; o__Clostridiales; f__Clostridiaceae; g__; s__	2.77
**175,469**	k__Bacteria; p__Firmicutes; c__Clostridia; o__Clostridiales; f__; g__; s__	2.7
**196,689**	k__Bacteria; p__Firmicutes; c__Clostridia; o__Clostridiales; f__; g__; s__	2.68
**371,647**	k__Bacteria; p__Bacteroidetes; c__Bacteroidia; o__Bacteroidales; f__S24-7; g__; s__	2.64
**437,151**	k__Bacteria; p__Firmicutes; c__Clostridia; o__Clostridiales; f__; g__; s__	2.64
**766,723**	k__Bacteria; p__Firmicutes; c__Clostridia; o__Clostridiales; f__; g__; s__	2.54
**944,987**	k__Bacteria; p__Proteobacteria; c__Gammaproteobacteria; o__Enterobacteriales; f__Enterobacteriaceae; g__; s__	2.49
**467,820**	k__Bacteria; p__Firmicutes; c__Clostridia; o__Clostridiales; f__; g__; s__	2.36
**264,909**	k__Bacteria; p__Firmicutes; c__Clostridia; o__Clostridiales; f__; g__; s__	2.36
**162,576**	k__Bacteria; p__Firmicutes; c__Clostridia; o__Clostridiales; f__; g__; s__	2.29
**276,761**	k__Bacteria; p__Firmicutes; c__Clostridia; o__Clostridiales; f__Ruminococcaceae; g__; s__	2.27
**229,452**	k__Bacteria; p__Firmicutes; c__Clostridia; o__Clostridiales; f__; g__; s__	2.26
**1,106,614**	k__Bacteria; p__Firmicutes; c__Clostridia; o__Clostridiales; f__; g__; s__	2.22
**338,317**	k__Bacteria; p__Firmicutes; c__Bacilli; o__Lactobacillales; f__Lactobacillaceae; g__Lactobacillus; s__	2.17
**380,753**	k__Bacteria; p__Firmicutes; c__Clostridia; o__Clostridiales; f__; g__; s__	2.16
**259,771**	k__Bacteria; p__Firmicutes; c__Clostridia; o__Clostridiales; f__; g__; s__	2.14
**308,900**	k__Bacteria; p__Firmicutes; c__Clostridia; o__Clostridiales; f__; g__; s__	2.14
**263,705**	k__Bacteria; p__Firmicutes; c__Clostridia; o__Clostridiales; f__Peptococcaceae; g__; s__	2.13

**185,923**	k__Bacteria; p__Firmicutes; c__Clostridia; o__Clostridiales; f__Ruminococcaceae; g__; s__	2.12
**1,108,306**	k__Bacteria; p__Firmicutes; c__Clostridia; o__Clostridiales; f__Ruminococcaceae; g__; s__	2.11
**1,059,824**	k__Bacteria; p__Firmicutes; c__Bacilli; o__Lactobacillales; f__Streptococcaceae; g__Streptococcus; s__	2.01
**376,462**	k__Bacteria; p__Bacteroidetes; c__Bacteroidia; o__Bacteroidales; f__S24-7; g__; s__	2.01

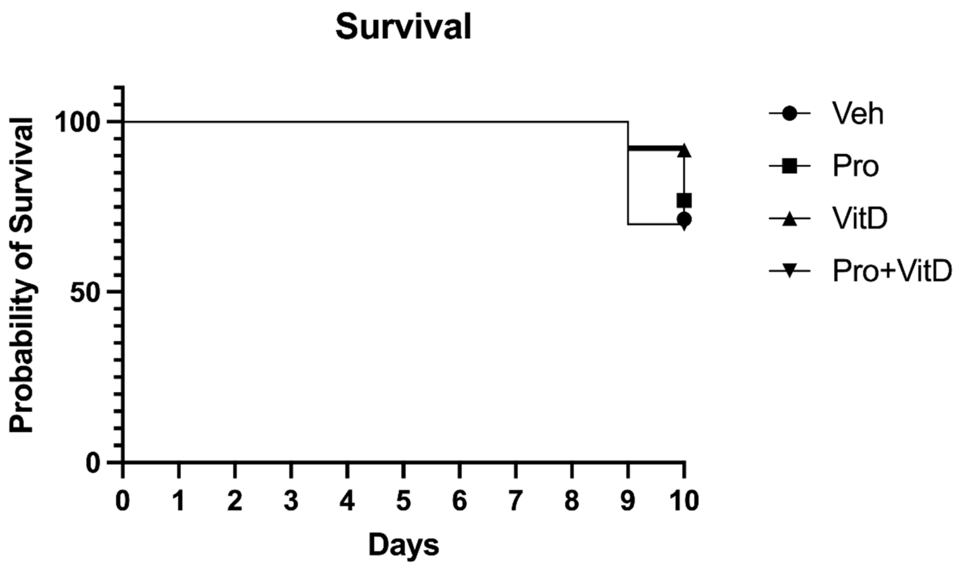

Supplementary Figure S1

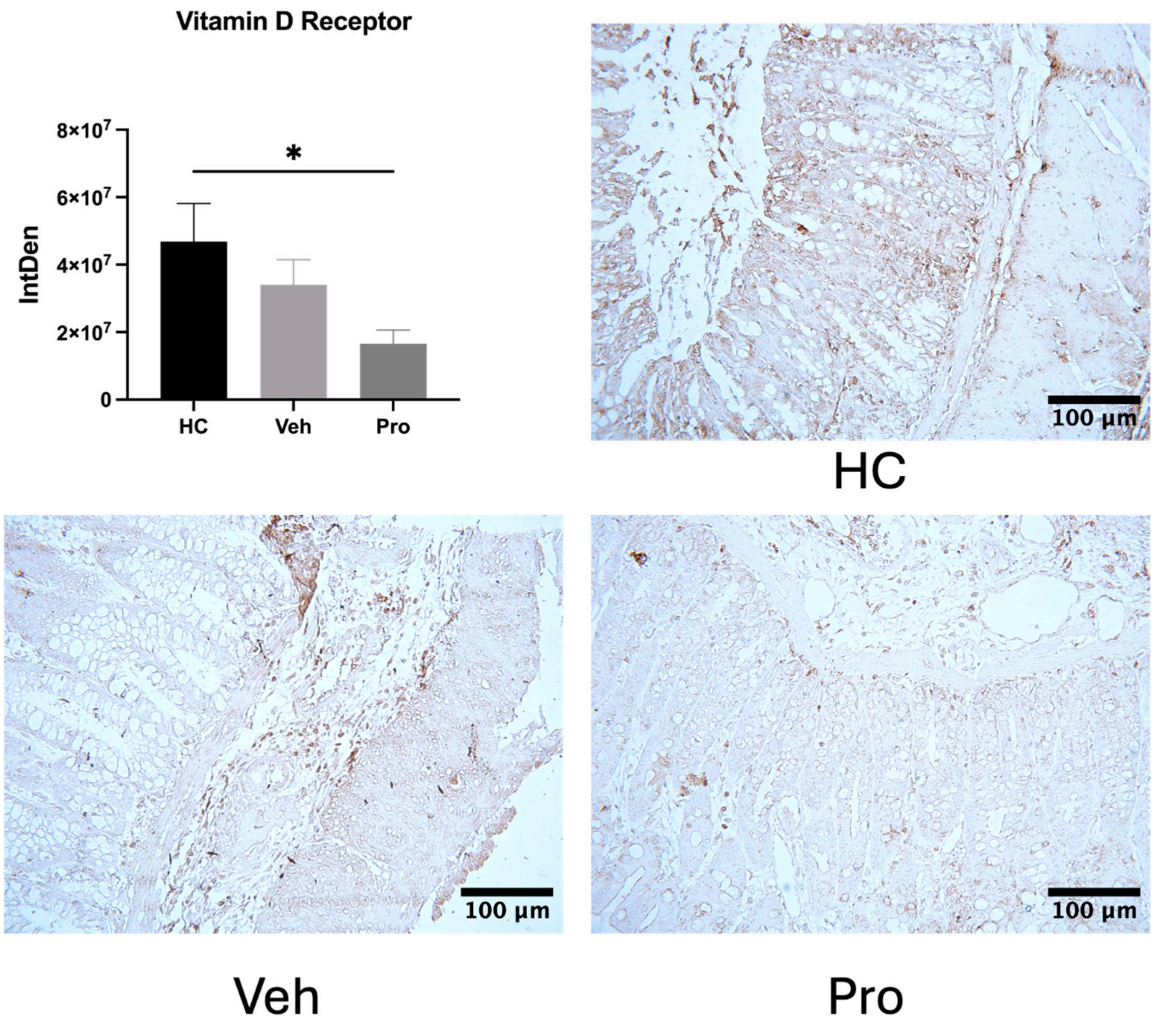

Supplementary Figure S2
